# Stress Biomarkers in Pigs: Current Insights and Clinical Application

**DOI:** 10.3390/vetsci11120640

**Published:** 2024-12-10

**Authors:** Vasileios G. Papatsiros, Georgios Maragkakis, Georgios I. Papakonstantinou

**Affiliations:** Clinic of Medicine, Faculty of Veterinary Medicine, School of Health Sciences, University of Thessaly, Trikalon 224, 43100 Karditsa, Greece; vpapatsiros@vet.uth.gr (V.G.P.); maragkakisg@gmail.com (G.M.)

**Keywords:** pigs, stress, biomarkers, animal welfare

## Abstract

Our study aimed to contribute to the understanding of the stress process in pigs to better assess and control their stress levels. Nowadays, pigs in intensive farming are exposed to several stress factors, such as weaning, transportation, diseases and vaccinations. As a result, the animals experience significant stress responses and inflammatory reactions that affect their health, growth and productivity. Therefore, it is crucial to assess their stress levels, and the use of stress biomarkers could be useful in their evaluation. An up-to-date overview of the different biomarkers that can be used for the assessment of stress is given. It also discusses the methods used to investigate these biomarkers, particularly non-invasive approaches, such as saliva sampling, as practical tools for monitoring animal welfare. In conclusion, our study highlights the importance of using multiple biomarkers for a comprehensive evaluation of stress and points to the need for further research to standardize the sampling procedures and improve stress management in pig farming.

## Simple Summary

This study highlights the various stress factors that pigs are exposed to on intensive farms and that affect their health, growth performance and productivity. Therefore, stress biomarkers should be used to monitor and control the stress levels of the animals. An up-to-date overview of the different biomarkers that can be used to assess stress is provided. In conclusion, our review emphasizes the need for further studies focusing on a comprehensive understanding of the stress process to better evaluate and control it.

## 1. Introduction

In commercial intensive pig farming systems, pigs are exposed to various stress factors, including diseases and vaccinations, weaning, transportation, gestation and farrowing, all of which trigger significant stress responses and inflammatory reactions in their bodies [1,2]. Inappropriate welfare standards further contribute to physical and psychological distress on farms, where pigs endure sudden temperature fluctuations [3,4], inadequate housing management [5], rough handling [6] and overcrowding [5,6]. In response to these stressors, pigs’ bodies produce a range of biomarkers, such as reactive oxygen species (ROS) [7], malondialdehyde (MDA) [8], cortisol and immunoglobulins (Ig) [2]. However, the same stress factor can trigger different reactions depending on the animal’s age, genetic background, farming system or previous exposure to the stress factors [2]. Although several animal welfare studies have already been carried out, the relationship between stress in pigs and its effects on their welfare has not yet been sufficiently researched. Further studies are therefore required. This paper provides a comprehensive overview of stress biomarkers in swine medicine, focusing on their clinical use to monitor and control stress in pigs. It highlights the importance of non-invasive methods of assessing stress to help improve animal welfare standards and management practices in intensive pig farming. Ultimately, this study highlights the need for further research to refine stress assessment methods and establish standardized protocols to alleviate stress-related problems in pigs.

In recent decades, increasing attention has been paid to animal stress on intensive pig farms due to its negative impact on pigs’ normal physiology, welfare and overall productive performance. From a practical standpoint, the classification of stress is based on its duration and causes. Stress can be acute (short-term, duration of minutes or several days) or chronic (long-term, duration of weeks, months or even years). Additionally, based on its cause, stress can be classified as follows [2]:

Social Stress: Pigs are often mixed in groups with unfamiliar mates at various production stages, such as during gestation, post-weaning or fattening stage, or before transport to slaughter. Under these housing conditions, pigs may perform aggressive behavior to exert and maintain dominance hierarchies, which induces stress [9]. Social stress can be acute, occurring immediately after group mixing, or chronic, experienced by socially subordinate or isolated animals [10] or because of repeated group mixing [11]. The level of social stress can vary based on factors like group size, the available space and the pigs’ gender and genetics. Larger groups tend to experience less social stress than smaller ones, as the likelihood of resource monopolization decreases with increasing group size [12]. In intensive housing conditions, the reduced space per animal leads to stress due to limited movement and restricted access to feed [13]. Studies reported that social interactions and aggressive behavior increase in sows as space allowance is reduced in group housing conditions [14], and growth performance declines linearly as space per pig decreases [15]. In addition, males tend to have a stronger response to social stress compared to females [14,15]. Individual differences in aggressive behavior following group mixing have a genetic background, with some pig genetic lines exhibiting less fighting when unfamiliar individuals are mixed [16]. Normal responses to stress, such as avoiding threats, confronting them or hiding, are considered adaptive behaviors. In contrast, stereotypies are considered abnormal and can occur in response to social stress, in addition to other causes, such as environmental factors (lack of space) [17]. Additionally, tail and ear biting are classified as abnormal behavior and are mainly influenced by nutritional deficiencies or high stocking density, but they can also be triggered by social stress factors, such as competition and aggression within the group [18].

Environmental stress: The control of various environmental factors (e.g., temperature, humidity, lighting, dust and gas concentrations, ammonia levels and sound intensity) is crucial under commercial conditions in intensive pig farming [19,20,21]. However, maintaining optimal environmental conditions is not always possible, which can lead to stress in pigs. This may occur in extremely hot or cold periods, on farms situated in noisy areas or during equipment malfunctions. The thermal environment’s impact on pigs depends not solely on ambient temperature but on the “effective temperature”, which considers factors like ventilation, floor type and ambient temperature. Additionally, inappropriate environmental conditions negatively affect animal welfare. The lack of bedding materials and slatted floors restrict pigs from exhibiting their natural behaviors [22]. This inability to perform highly motivated behaviors can result in a stress response, such as sows being prevented from engaging in nest-building behavior before farrowing experience stress [23].

Metabolic stress: This stress is usually caused by feed and/or water restriction or deprivation. It is possible for metabolic stress to appear under commercial pig farming conditions, such as sows in the gestation stage, due to restricted feeding, resulting in chronic stress of hunger [24]. For example, it is usual for the more submissive sows to have less or no access to feed under group housing conditions [25].

Immunological stress: Immunological stress can be caused by the challenge of various pathogens occurring during disease occurrence or after the administration of vaccines [26]. In addition, stress is not only considered a possible cause but also a result of infectious diseases. Under stress conditions, changes have been noticed in white blood cell count (e.g., leukocytes), mitogen-induced cell proliferation, natural killer cell cytotoxicity and circulating inflammatory markers, resulting in increased susceptibility of animals to various infectious agents [27]. In clinical cases of an existing disease, stress can be an important predisposing factor for the emergence of a different disease, further complicating the clinical picture [15].

Stress from animal handling: The routine handling management practices under commercial pig farm conditions can cause acute stress in pigs. Common routine practices in commercial farms, such as restraint or blood sampling, can cause high stress, whereas others (e.g., tattooing) can induce more moderate stress [28]. Similarly, the practice of castration in piglets without anesthetics or analgesics, which is a common practice on some farms, can cause serious stress [29].

## 2. Stress Conditions in Intensive Pig Farming

Weaning and transport are considered some of the most important stress factors in intensive pig farming systems. These production stages are characterized by significant challenges that lead to a high degree of stress.

Weaning is very stressful for piglets, as they are separated from their mother and littermates, transported to a new housing environment, changed from a warm liquid diet (milk) to solid feed and co-mingled with non-littermates in large groups, thus increasing their exposure to pathogens, dietary or environmental antigens, as well as to various health care practices (e.g., vaccination and antibiotics administration) [30]. Typically, piglets are separated from the sow abruptly at weaning and mixed in groups per pen until they reach 30 kg of body weight [31]. This practice induces stress due to sudden physiological, environmental and social changes [30]. The indicators of this stress include low feed intake and lower growth performance, increased levels of cortisol in saliva and plasma, agonistic-behavior-inducing skin lesions and an increased risk of abnormal behaviors and post-weaning diarrhea [32].

The implementation of Council Directive 2001/88/EC in 2001 marked a significant shift in pig farming across the European Union, transitioning from the housing of sows in individual gestation stalls to group housing. This trend is reflected globally in pig-producing regions, such as the United States [33,34,35]. Group housing is considered more welfare-friendly, as it offers sows greater freedom of movement and opportunities for social interactions compared to individual gestation stalls [36]. However, in group housing of sows, increased aggression among sows due to competition for limited feed, social conflicts from continuous re-mixing and the subordination or isolation of some individuals are noted. Additionally, suboptimal physical environments may contribute to long-term (chronic) stress [2]. Numerous studies have examined the types and physiological effects of acute stressors that sows experience, such as mixing unfamiliar individuals, which leads to increased aggressive behavior of sows to establish and maintain a dominance hierarchy. This mixing is highly stressful and is associated with increased heart rate, increased levels of catecholamines in plasma, as well as elevated cortisol levels [37]. Mixing unfamiliar pigs is also a significant stress factor for weaning, growing and finishing pigs, with evidence showing profound physiological and behavioral changes following mixing [38,39]. Overall, research confirms that mixing is a particularly stressful event [38]. Other examples of acute stress factors include transportation, social isolation and physical restraint [40,41].

Transport is another significant stress factor in pig production, as animals are exposed to various stressors, including separation from familiar surroundings, truck loading and unloading, fasting, environmental changes (temperature and humidity), noise, vehicle vibrations and high stocking density [42]. Increased cortisol levels, heart rate and a suppressed immune response are among the physiological effects of transport stress. In addition, it not only affects animal welfare but also has a negative impact on meat quality, for example, by increasing the incidence of pale, soft and exudative meat (PSE) [42]. The guidelines for reducing transport stress recommend avoiding extreme temperatures and overcrowding, minimizing the time spent on the truck, using non-slip flooring on loading ramps and ensuring proper animal handling by qualified personnel [43].

Slaughter also introduces a critical phase in which pigs are exposed to additional stress factors, which, if poorly managed, can severely affect animal welfare. Stunning methods (electric or gas) are a crucial factor in stress levels, as inappropriate stunning can lead to unnecessary suffering [42]. Furthermore, suboptimal housing conditions, such as poor ventilation, or long waiting times exacerbate stress responses, including increased cortisol production and restlessness. These stress factors are not only ethically questionable but also contribute to poor meat quality [42]. Introducing better handling methods, improving barn conditions and ensuring effective stunning are crucial to minimizing stress at this stage.

## 3. Consequences of Stress for Pig Production

In commercial intensive pig farming systems, pigs are exposed to long-term and short-term stressors, which significantly impact their welfare. High stress levels and poor welfare conditions negatively affect five key areas of pig production: growth performance, reproduction, behavior, immunity and meat quality [44]. The severity of these effects varies depending on the duration and intensity of the stress, as well as the animal’s early experiences, age and genetic background [45].

Stress is usually associated with decreased growth performance parameters (e.g., feed intake, daily weight gain and body weight) [46]. In boars, stress has been reported to reduce both ejaculate volume and semen quality. In breeding stock, stress is associated with a decreased number of piglets per litter, increased irregular returns to estrus, longer weaning-to-estrus intervals and lower fertility rates, which consequently decrease farm productivity [47]. Stress also impairs the immune function, leading to reduced immune responses and even suppression of vaccination efficacy [27]. This reduced immunity increases the susceptibility of animals to diseases, increasing production costs and therefore reducing farm productivity [48].

Moreover, stress adversely affects meat quality, contributing to higher instances of pale, soft and exudative (PSE) meat and dark, firm and dry (DFD) meat. Handling systems that involve stressful conditions are associated with decreased meat quality.

**Figure 1:**
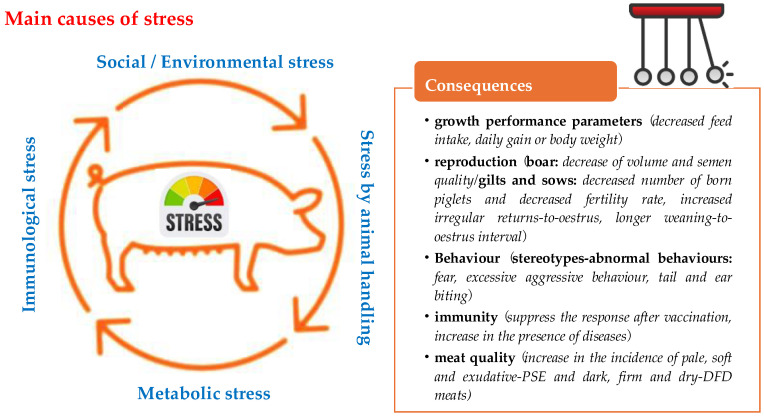
Main causes and consequences of stress in pigs.

Figure 1 summarizes the primary causes of stress in pigs and their consequences, including behavioral abnormalities and impacts on meat quality.

## 4. Stress Biomarkers in Pigs

Biomarkers can provide valuable insights into the physiological responses to stress in animals and are useful for assessing animal welfare and stress levels [49]. Numerous studies have evaluated a variety of biomarkers in the saliva and blood of pigs, as summarized in Table 1.

### 4.1. Metabolic and Behavioral Stress Biomarkers

Glucocorticoids can induce oxidative stress under experimental conditions, while their role has been extensively studied in animals [50]. Cortisol, as a primary glucocorticoid, is a common stress biomarker for pigs. However, cortisol levels can be influenced by several factors (e.g., circadian rhythm and genetic background), which may limit its reliability as a stress biomarker [51]. For instance, morning cortisol levels can be >40% higher than afternoon levels, although an afternoon peak has been reported [52]. Moreover, cortisol levels seem to decrease with age and vary by gender (e.g., 15% higher levels in barrows in comparison to gilts) [39].

Plasma or serum have been the most common matrices for cortisol measurement in pigs, as they reflect both protein-bound and free cortisol, which is the biologically active fraction [39]. However, recently, there has been a growing preference for using saliva because it can be collected non-invasively and only reflects free cortisol [18,53]. Other useful stress biomarkers include cortisone and alpha-amylase, especially under farm conditions [54,55]. Recently, oral fluids have been proposed as an alternative to blood for stress biomarker measurement, as they reflect real-time biomarker levels without the stress associated with invasive sampling practices [50]. The oral fluid contains stress biomarkers that are suitable for monitoring stress impacts and offer several advantages: non-invasive method, easy to collect, cost-effective and no adverse effects on animal health and welfare [55]. In addition to saliva, cortisol can also be measured in other matrices, such as urine, feces or hair, which provide insights into average cortisol concentrations over hours (urine, feces) or weeks (hair) [56,57].

### 4.2. Immune Stress Biomarkers

Acute phase proteins (APPs), such as pig major acute phase protein (Pig-MAP), haptoglobin (Hp), C-reactive protein (CRP) and serum amyloid A (SAA), have been reported as stress biomarkers in pigs in several studies [40]. APPs, which are blood proteins, are synthesized by the liver and released into the systemic circulation to restore the organism’s homeostasis [52]. Their concentration is modified in response to inflammation, infection and physical or psychological stress. In addition, APPs could be a tool for disease diagnosis in pigs, especially to detect acute inflammation, measuring in plasma and saliva [40,58]. The increase in APP levels, such as Pig-MAP, Hp, CRP and SAA, has been reported after long and short transportation, as well as following isolation and changes in the feed administration [40]. Studies have reported measurement of APPs in pigs under stressful conditions, but there is no clear evidence of APPs response in stressful conditions, and contradictory results have been reported. For instance, while Hp levels increased after 20 min of transport and 3 h of lairage, 45 min of transport or social isolation resulted in no significant alterations [40,59]. In addition, SAA is increased in isolated animals but does not change after exposure to stress due to changes in feed administration [40].

Non-invasive biomarkers, such as testosterone, chromogranin A (CgA) and immunoglobulin A (IgA), provide valuable insights into various physiological responses to stress [60]. Testosterone levels have been observed to increase during acute stress, such as restraint or short-term road transport, indicating a possible role of adrenal androgen production during stress [61]. Immunoglobulin A reflects changes in mucosal immunity and has been shown to increase following stressors such as isolation or endotoxemia [62,63,64]. In contrast, chromogranin A, which can be used as a marker for sympathetic-adrenal system (SAM) activation, has been shown to be a stable indicator that is unaffected by variables such as age, gender or circadian rhythms [64,65]. This makes it particularly useful for the assessment of stress under different conditions, such as restraint, food deprivation or social isolation. While all these biomarkers provide a holistic approach to stress, reflecting endocrine, neurological and immunological system responses, their non-invasive sampling methods make them practical tools for monitoring welfare on farms.

### 4.3. Oxidative Stress Biomarkers

Several stress biomarkers have been identified in pigs, including total antioxidant capacity (TAC), glutathione (GSH), catalase activity (CAT), protein carbonyls (CARB), thiobarbituric acid reactive species (TBARS), oxidized glutathione (GSSG), thiol proteins, glutathione peroxidase (GPx), malondialdehyde (MDA) and reactive oxygen metabolites (ROM) [66,67,68,69,70]. Oxidative stress biomarker changes in pigs can arise in various situations throughout their productive period, correlating with fluctuations in the animals’ oxidant balance. For instance, reactive oxygen species (ROS) levels increase during transport, resulting in oxidative stress [71]. The weaning period has also been associated with increased oxidative stress and an increase in ROS levels [71]. Furthermore, the serum levels of ROS and TBARS are elevated during late gestation and early lactation in comparison to early gestation [72]. In addition to the previously mentioned stress biomarkers, TAC assessments, such as the ferric-reducing ability of plasma (FRAP), have been evaluated in the pig serum, particularly in field studies with feed supplements [73]. Another TAC assay is the Trolox equivalent antioxidant capacity (TEAC), which can assess TAC in the plasma of piglets under post-weaning stress conditions [74].

In addition, serotonin levels may also serve as an indicator of physiological stress in stress-sensitive species such as pigs, complementing conventional biomarkers of oxidative stress, such as thiobarbituric acid reactive substances (TBARS), protein carbonyls (CARBS) and total antioxidant capacity (TAC). Bruschetta et al. (2024) [75] observed significant interactions between serotonin and oxidative stress parameters in dogs. Their study emphasized the role of serotonin in buffering oxidative damage under acute stress conditions, highlighting its potential as a biomarker of stress and well-being in veterinary medicine [75]. Serotonin may also be correlated with neurological and systemic stress responses, as exercise has been shown to modulate serotonergic activity in leukocytes, highlighting its role in systemic stress response and immune regulation [76].

While several studies have investigated biomarkers such as cortisol and acute phase proteins and provided a wealth of data, other stress biomarkers remain under-researched. This disparity in the research landscape inherently limits the depth of discussion on some markers. The unbalanced attention given to different stress markers reflects the current state of research in the field. For some biomarkers, such as cortisol and acute phase proteins, extensive studies have been conducted, providing a wealth of data and insights that allow us to examine them in more detail. However, for other biomarkers, the available literature is less extensive, limiting the depth of discussion we were able to provide. This disparity in reporting should not be used to favor certain biomarkers over others but rather to present the results appropriately based on the available data. Future studies should aim to bridge this gap to ensure a more comprehensive understanding of stress responses in pigs.

Table 1 summarizes the main biomarkers of stress in pigs, highlighting the results of specific studies and their relevance to the different physiological systems they primarily reflect.

**Table 1:** Stress biomarkers in pigs, their primary physiological system, biomatrices and important findings.

Biomarker *	Physiological System	Biomatrices	Observations	References
SAA	Immune System	Saliva Blood (serum)	Increased levels after 1 min of restraint stress Increased levels in pigs isolated for short periods Increased levels of physical restraint Increased levels after short- or long-duration road transport	[40,77]
Hp	Immune System	Blood (serum)	Increased levels after 20 min of transport and 3 h of lairage Increased levels after long-duration transport	[59]
Cortisol	HPA Axis and Endocrine System	Saliva Blood (serum)	Increased levels in pigs isolated for short periods Increased levels after short-duration road transport	[40]
Pig-MAP	Immune System	Blood (serum)	Increased levels after short- or long-duration road transport	[58,78]
CRP	Immune System	Blood (serum)	Increased levels after short- or long-duration road transport	[58]
CgA	Sympathetic-Adrenal-Medullary System	Saliva	Increased levels after restraint with a nasal snare Increased levels after refeeding following a period of feed deprivation Increased levels in pigs after isolation or group mixing	[64,65]
TBARS	Oxidative Stress System	Blood (plasma)	Higher levels during late gestation (days 90 and 109) and early lactation (days 1 and 3) vs. early gestation (day 10) Increased levels in pigs under heat stress	[69,72]
CARBS	Oxidative Stress System	Blood (plasma)	Increased levels in pigs under heat stress	[69]
IgA	Immune System	Saliva	Increased levels in pigs with restraint stress or after isolation	[62,64]
IL-18	Immune System	Saliva	Increased levels during 60 min acute immobilization stress	[62]
Testosterone	HPA Axis and Endocrine System	Saliva	Increased levels after restraint with a nasal snare or after short-duration road transportation	[64]
Serotonin	Oxidative Stress System	Blood (serum)	Increased levels after road transport Increased levels after physical exercise	[77,79]

* Serum amyloid A (SAA), haptoglobin (Hp), pig major acute phase protein (Pig-MAP), C-reactive protein (CRP), chromogranin A (CgA), thiobarbituric-acid-reactive substances (TBARS), protein carbonyl (CARB), immunoglobulin A (IgA), interleukin-18 (IL-18).

## 5. Sampling for Stress Biomarkers in Clinical Practice

Stress biomarkers in pigs have been assessed using serum or plasma samples. However, blood sampling can be very stressful for pigs due to the required immobilization, and the impact of this stress on various oxidant biomarkers is not well understood. In contrast, saliva offers several advantages over blood as a biological fluid; it is easy to collect without inducing stress [50]. Recently, saliva has been increasingly recognized as an accessible source of biomarkers in pigs that can provide insights into animal health and welfare [80]. Furthermore, studies have shown that saliva is a suitable sample for health monitoring and disease diagnosis [81].

Although the information regarding oral fluid collection is limited, it typically employs low-cost absorptive devices like cotton ropes or sponges [82]. Oral fluid collection is simple, inexpensive, quick and non-invasive, making it an effective method for assessing the health and welfare status of pigs. However, the type of collection device and the sample processing methods (e.g., centrifugation and filtration) can influence the results of oral fluid testing [83]. Other studies have underscored the negative impact of sample contamination on test performance [84]. Therefore, there is a need for further standardization of the collection methods to facilitate their routine use in veterinary practice. While cotton ropes are commonly used for collecting oral fluid for pathogen detection, they may not be the best choice for biomarker analysis due to the high-risk contamination [84]. Stress biomarkers in porcine oral fluid exhibited variability based on the collection material and animal age, which are important factors for consideration in the interpretation of results. From a practical standpoint, sponge sampling provides the best combination of reduced sampling time and low-risk contamination. Unlike ropes, which do not require constant supervision from an operator, veterinarians often need to enter the pen to position the ropes optimally, making sponges more convenient [85].

The analysis of saliva can measure various biomarkers, including cortisol, SAA, CgA, total esterase, oxytocin, A, ADA and Ig. Cortisol, a specific biomarker, can also be detected in hair, urine and feces, with urine samples allowing for the analysis of catecholamines as non-invasive indicators of animal welfare [56,57,86]. Moreover, in addition to saliva, analyzing welfare biomarkers in other non-invasive matrices (e.g., urine, feces, milk and hair) is associated with less or minimal acceptable pain and stress. Additionally, the avoidance of blood collection (considered invasive) encourages animal welfare, as each blood sampling is classified as a procedure under Directive 2010/63/EU and thus requires authorization.

## 6. Clinical Practice: Behavioral or Health Disorders and Stress Biomarkers

Various social and environmental conditions, such as dominance struggles or frequent regrouping, can trigger stress reactions in piglets that lead to behavioral disorders. These disorders, such as tail biting or aggression, can lead to inflammation or tissue damage [64]. Therefore, the evaluation of stress biomarkers could be a valuable tool to monitor behavioral stress in pigs. Previous studies have reported that the determination of salivary cortisol and chromogranin A (CgA) levels could contribute to the detection of acute stress responses due to aggression, social isolation or confinement. Elevated cortisol levels may indicate stress due to social mixing, while CgA could provide information about sympathetic nervous system activation that may be triggered by social stressors [64,65]. In addition, acute phase proteins, such as serum amyloid A (SAA) and haptoglobin (Hp), have been shown to signal chronic stress resulting from persistent behavioral disturbances [40,59,75]. If these biomarkers are routinely measured, stress-inducing factors in the environment can be detected early, and timely management measures, such as adjusting group size, improving environmental conditions or changing feeding strategies, can be facilitated.

The negative impact of various stress factors on immunity has been previously documented [87,88]. This impact can manifest as either a mild response or an acute clinical reaction that lasts for a short period. Furthermore, a higher incidence of disease in “stressed” pigs has been identified as a potential indicator of decreased welfare due to compromised immunity [87]. Conversely, the long-term effects of stress, such as immunosuppressive or immunomodulatory functions, appear to be associated with chronic conditions. Chronic stress may lead to alterations in innate immunity, resulting in leukocytes migrating through the endothelial lining to infiltrate inflamed areas, which can cause a reduction in blood leukocyte counts [89].

To assess the effects of stress on adaptive immune responses, lymphocyte proliferation is frequently examined [88], with studies indicating that repeated stress models suppress lymphocyte proliferation [90]. Chronic psychological stress seems to accelerate biological ageing, with oxidative damage being a significant potential mediator in this process [91]. Free radicals serve to combat invading micro-organisms but can also result in tissue damage during inflammation [92]. Evidence of oxidative stress has been noted in various infectious farm animal diseases [93]. Inflammation, a key mechanism of innate immunity, must be carefully regulated to ensure it remains beneficial to the host [94]. The immune system’s activation triggers the release of cytokines that stimulate the hypothalamic–pituitary–adrenal (HPA) axis, thereby increasing peripheral glucocorticoid levels [95].

The link between stress and disease has been well established [87]. Numerous studies have documented the relationship between abnormal animal behaviors—such as excessive aggression, vocalizations or prolonged periods of inactivity—and stress, alongside an increased risk of disease or variations in immune marker levels following different experimental stressors [40,49]. However, fewer studies have examined the changes in immune markers under stressful field conditions in pigs, and even fewer have compared the behavior of stress markers concerning field diseases.

It has been reported that different stress factors induce distinct physiological stress responses in pigs, as diseased pigs may exhibit increased biomarker levels related to the HPA axis or the autonomic nervous system, depending on the pathological damage. Despite these variations in the activation levels of different host responses, overall positive correlations have been noted between psychological stress and immune biomarkers [49]. The interplay between oxidative stress and inflammatory reactions has also been investigated in various infectious pig diseases (e.g., pneumonia, enteritis and sepsis) [93]. A recent study reported a correlation between salivary oxidative stress and immune biomarkers, but variations based on the analyzed pathological condition were also noticed [96]. Moreover, oxidative stress can be induced by immune activation, physical exercise or stress [97], with stressors potentially exacerbating oxidative stress reactions [92].

In pigs, most research works have concentrated on the response to respiratory diseases, identifying four APPs: SAA, Hp, CRP and Pig-MAP (Table 2).

A comprehensive understanding of the stress process is essential for reducing and controlling stress levels in pigs. A valuable contribution in this direction could be the combination of biomarkers for stress assessment in pigs, which would integrate indicators from different physiological systems. In particular, combining immune biomarkers such as SAA and Hp with markers of oxidative stress such as TBARS or CARBS could provide further data into the interaction between inflammation and oxidative damage under stress conditions. Likewise, combining the assessment of cortisol levels—a key hormone reflecting activation of the hypothalamic–pituitary–adrenal (HPA) axis—with neuromodulators such as serotonin or CgA could help distinguish between acute and chronic stress and reveal stress-related behavioral and physiological changes. By integrating these biomarkers, tailored to specific stressors, such as transport, heat or social mixing, a more accurate and holistic assessment of animal welfare can be achieved, allowing for timely interventions and improved management practices.

## 7. Conclusions

In recent years, society’s interest in compliance with animal welfare standards for pigs has increased. In this context, the stress to which animals are exposed in intensive pig farming is being given serious consideration because of the associated effects. In addition to the consequences for animal welfare, it can also lead to problems with growth performance, reproductive disorders, poor immune response and deterioration in meat quality. Due to the various causes that can trigger stress and the different physiological systems involved in the stress response, a variety of biomarkers should be used to assess stress. A comprehensive understanding of the stress process is essential for reducing and controlling stress levels in pigs. Therefore, it is crucial to integrate the analysis of stress biomarkers into farm management practices to identify and mitigate stress factors, such as suboptimal housing conditions, social conflicts and transportation problems. A multi-biomarker approach can better capture the complexity of stress responses in pigs and thus improve welfare monitoring and health interventions. The results presented in this paper could serve as a basis for the development of comprehensive animal welfare protocols and emphasize the importance of collaboration between researchers and industry stakeholders. Further studies are needed to standardize biomarker thresholds and validate their clinical applicability in different production systems, so that pig farming can progress in both animal welfare and productivity.

## Figures and Tables

**Table 2:** Stress biomarkers related to health and behavioral disorders or pathogens in pigs.

Health–Welfare Disorders or Pathogens *	Stress Biomarker **	Age	Biomatrices	References
Swine dysentery (*Brachyspira hyodysenteriae*)	Increased Hp and SAA levels	Weaners Growers Finishers	Serum	[98]
Porcine pleuropneumonia (*App*)	High Hp, CRP and Pig-MAP levels, 4–5 days before the development of specific antibodies Strongly induced SAA response	Growers	Serum	[99]
	Hp, CRP and Pig-MAP response to App-infected pigs	Growers	Serum	[100]
*S. suis*	Increased SAA, oxytocin, Hp and AOPP in infected animals Increased levels of CK, lactate, aspartate aminotransferase, lactate dehydrogenase, ADA, procalcitonin and aldolase in infected animals	Growers	Saliva	[101]
	Fast-increased CRP and SAA concentrations Acute clinical and subclinical *S. suis* infection: at least 1 or >1 measurement of CRP, SAA, Hp, pig-MAP and Apo A-I	Weaners	Serum	[102]
*P. multocida*	Elevated Hp concentration until the end of the study Increased SAA and Pig-MAP concentration 3–7 days dpi	Weaners	Serum	[103]
PCV2 (PMWS)	Higher Pig-MAP and Hp concentrations in PMWS-affected pigs	Weaners Growers	Serum	[104,105]
	PCV2-infected piglets with severe PMWS: increased levels of CRP and IL-10	Weaners	Serum	[106]
PRRSV	Detection of Hp, CRP and Pig-MAP at 0–21 dpi PRRSV infection Heterogeneous and dependent Hp, CRP and Pig-MAP response with viral strain Correlation of Hp, CRP and Pig-MAP response with the severity of clinical signs Hp: most sensitive biomarker for PRRSV infection Distinction between PRRSV-infected and non-PRRSV-infected pigs: use Hp and CRP, but not Pig-MAP	Weaners	Serum	[80]
	Higher Hp and CRP concentrations in both saliva and serum samples from PRRSV-infected pigs	Weaners Growers Finishers	Saliva Serum	[107]
	Increased SAA levels in saliva and serum in diseased pigs	Growers	Saliva Serum	[108]
	Higher activities of SOD in PRRSV-positive weaners and finishers Higher *GPx* activities in PRRSV-positive weaners Lower *GPx* activity in PRRSV-positive finishers Higher concentrations of serum total protein in PRRSV-positive weaners and finishers	Weaners Growers Finishers	Serum	[109]
	Increased Hp and Pig-MAP levels at 10 dpi Delayed and highly variable increase in CRP and SAA	Weaners	Serum	[110]
	Increased Hp levels at 7–21 dpi in the infected pigs Increased IL-6 in the infected pigs	Weaners	Serum	[111]
SIV	Increased CRP and Hp levels, with peak 24 h later than the cytokines	Weaners	Serum	[112]
	Increased Hp and SAA levels after SIV infection CRP and Pig-MAP concentrations remaining unchanged	Weaners	Serum	[113]
ASFV or CSFV	Increased Hp, CRP and SAA levels in ASFV- and CSFV-infected pigs Most noticeable increased SAA concentration, followed by CRP and then Hp	Growers Finishers	Serum	[114]
Co-infection with *M. hyo* and SIV (H1N1)	Higher levels of ROM 3 weeks after infection with *M. hyo* Increased Hp levels at 2 H1N1 dpi Increased *GPx* levels	Weaners	Serum	[115]
Co-infection with swine influenza virus (H1N1) and *Haemophilus parasuis*	Increased concentrations of SAA, Pig-MAP, Hp and CRP at 3–7 dpi	Weaners	Serum	[116]
Co-infection with SIV (H1N1) and *P. multocida*	Increased CRP concentration at 1 dpi and remaining higher at 3 dpi Higher SAA levels at 2–3 dpi Elevated Hp levels at 3 dpi until the end of the study Pig-MAP at 3–7 dpi Increased CRP, Hp and SAA concentrations before the detection of specific antibodies Positive correlations between Hp and SAA concentration and lung scores Positive correlations between clinical score and Pig-MAP and SAA concentration	Weaners	Serum	[117]
Co-infection with SIV (H1N1) and *App*	Stronger response of APPs in *App*-inoculated groups vs. the SIV single-infected group Elevated levels of SAA from 1 to 2 dpi in all inoculated groups Pig-MAP levels remaining unchanged Increased Hp concentration in all inoculated groups Increased CRP concentration in App-inoculated pigs and App+H1N1N-inoculated pigs	Weaners	Serum	[118]
Co-infection with PRRSV, ADV, PCV2 and *M. hyo*	Higher CRP, SAA and Hp levels in PRRSV-infected pigs Higher Hp levels in ADV-affected pigs Higher CRP, SAA, Pig-MAP and Hp levels in PCV2-infected pigs Higher CRP, SAA, Pig-MAP and Hp levels in *M. hyo*-infected pigs	Weaners	Serum	[119]
Co-infection with ASFV and ADV	Correlation of high levels of Pig-MAP with the clinical course of ASF infection Increased Hp levels in ASF- and AD-infected pigs Increased CRP concentration in AD infection	Growers Finishers	Serum	[120]
Porcine respiratory disease complex (PRDC)	Higher Hp concentrations in PRDC-affected pigs Higher Hp concentration in pigs with PRDC vs. PMWS-affected pigs Lower CRP in pigs with PRDC vs. PMWS-affected pigs Increased CRP and SAA levels with the lymphoid depletion score Higher Hp levels in pigs with no or low depletion vs. pigs suffering severe lymphoid depletion		Serum	[121]
Vaccination at weaning against PCV2 and SIV, under heat stress	Decreased *GPx* activity Higher Hp concentrations and lipid peroxides	Weaners	Blood	[122]
*Sarcoptes scabiei* var. *suis*	Lower GSH, SOD, *GPx* serum levels in pigs suffering from clinical and subclinical sarcoptic mange Higher LPO serum levels in infected pigs Lower CAT, SOD and GST activities in the skin of the diseased pigs Higher LPO in the skin of the diseased pigs	1–2 years of age	Blood Skin	[123]
Post-partum dysgalactia syndrome (PDS)	Higher SAA and Pig-MAP concentrations in PDS-affected sows	Sows	Serum	[124]
Swine inflammation and necrosis syndrome (SINS)	SINS-affected pigs: increased CRP concentrations	Suckling Piglets Weaners	Serum	[125]
Stocking density	Increased Pig-MAP in higher density housed pigs	Growers	Serum	[126]
Tail biting	Decreased oxytocin levels in pigs with tail-biting lesions Increased procalcitonin levels in pigs with lesions	Weaners	Saliva	[127]
Endotoxemia, administration of LPS	Elevated cortisol, Hp, CRP and IgA activity in LPS-treated pigs	Growers	Saliva	[63]
Vaccine-associated enhanced respiratory disease following SIV infection	Elevated CRP, Hp and Pig-MAP in vaccinated pigs	Weaners	Serum	[128]
Heat stress	Increased TBARS and CARBS levels in sows suffering from heat stress	Sows	Plasma	[68]
Mycotoxicosis (aflatoxin B1, fumonisins: fumonisin B1 and fumonisin B2)	Increased TBARS and CARBS levels in sows fed with contaminated feed	Sows	Plasma	[69]
Mycotoxicosis (fumonisins)	Increased TBARS and CARBS levels in pigs fed with contaminated feed	Weaners	Plasma	[70]

* *Actinobacillus pleuropneumoniae* (App), *Streptococcus suis* (*S. suis*), *Pasteurella multocida* (*P. multocida*), *Porcine circovirus type 2* (PCV2), *Post-weaning multi-systemic wasting syndrome* (PMWS), *Porcine reproductive and respiratory syndrome virus* (PRRSV), *African swine fever virus* (ASFV), Classical swine fever virus (CSFV), *Mycoplasma hyopneumoniae* (*M. hyo*), *Swine influenza virus* (SIV), *Glaesserella* (formerly *Haemophilus*) *parasuis* (*G. parasuis*), *Aujeszky’s disease virus* (ADV), *Lipopolysaccharide* (LPS). ** Serum amyloid A (SAA), Haptoglobin (Hp), C-reactive protein (CRP), Pig major acute phase protein (Pig-MAP), Immunoglobulin A (IgA), Thiobarbituric-acid-reactive substances (TBARS), Protein carbonyl (CARB), Lipid peroxides (LPO), Glutathione (GSH), Superoxide dismutase (SOD), Glutathione peroxidase (GPx), Adenosine deaminase (ADA), Creatine kinase (CK), Advanced oxidation protein products (AOPP), Reactive oxygen metabolites (ROM), Negative APPs albumin and apolipoprotein (Apo) A-I, Days post-infection (dpi).

## Data Availability

No new data were created or analyzed in this study. Data sharing is not applicable to this article.
